# Resting State Heart Rate Variability in Depression: An Introductory Narrative Review of Cross-Sectional and Longitudinal Evidence

**DOI:** 10.3390/jpm16020087

**Published:** 2026-02-03

**Authors:** Evelien Van Assche, Carmen Schiweck

**Affiliations:** 1 Department of Psychiatry, University of Münster, 48149 Münster, Germany; evelien.vanassche@ukmuenster.de; 2 Department of Psychiatry, Psychotherapy and Psychosomatics, Goethe University Frankfurt, 60528 Frankfurt, Germany; 3 Cooperative Brain Imaging Center—CoBIC, Goethe University Frankfurt, 60528 Frankfurt, Germany

**Keywords:** heart rate variability, depression, longitudinal studies, treatment, biomarker

## Abstract

Cardiovascular health and depression influence each other bidirectionally and negatively, leading to high comorbidity rates, and favouring higher morbidity and mortality. Heart rate variability (HRV) has received much attention as a “biomarker” for major depressive disorder, with studies suggesting its potential both as a diagnostic and as a predictive biomarker. This narrative review offers a first orientation to the evidence base for researchers entering the field. We present and discuss the state-of-the-art evidence of cross-sectional and longitudinal studies (including observational, pharmacological interventions, and non-pharmacological interventions) linking depression and/or depressive symptoms to HRV by highlighting meta-analyses and key studies in the field. We briefly discuss the physiological context for interpretation of HRV and important confounders to consider, including the influence of genetics, age, sex, antidepressant medication, and lifestyle factors. Finally, with this information at hand, we discuss and provide guidance for factors to consider when using HRV in designing a study. Our literature review indicates that while there is potential for vagally mediated HRV to be of value in predicting future depression, more in-depth and stratified research of HRV is beneficial to the field and the understanding of what HRV can mean for depression research.

## 1. Introduction

Blaise Pascal’s quote “*We know the truth, not only by the reason, but also by the heart*” (Pensées, Section 282) is just one example that demonstrates what humans have understood intuitively: that there is a strong connection between the heart and brain. In the 20th and 21st century, this relationship was formally quantified. Using national registry and population-based data, it is now known that individuals with mental health disorders face a substantially greater likelihood of developing cardiovascular disease (CVD) and vice versa. As such, the 12-month prevalence rates of mental disorders in patients with CVD are more than 40% [1], with higher mortality rates and poorer overall outcomes [1,2]. To illustrate, depression increases the risk for incident coronary heart disease, and is associated with cardiovascular morbidity and mortality in patients diagnosed with coronary heart disease [3]. Conversely, the presence of CVD can also precipitate the new onset of mental health disorders [4].

This body of evidence has undoubtedly contributed to the rising focus on cardiovascular health and associated factors in psychiatry. To study the connection between the mind and the heart, researchers have, next to defining classical risk factors and ultrasound-guided explorations, focused on non-invasive techniques, such as electrocardiography (ECG), to explore the connection between the brain and the heart [5,6]. ECG-derived heart rate variability (HRV) has been studied both as a potential biomarker of stress sensitivity and for various psychiatric disorders, including depression [6,7]. In the context of mental health and in the age of precision medicine, prognostic and predictive biomarkers are of particular interest because they may enable early identification of individuals at heightened risk for depression or poor treatment response [8]. HRV is especially promising in this regard; it can be measured non-invasively and is readily accessible via wearable technology [9]. Given the increasing accessibility of physiological monitoring in everyday settings, clarifying whether HRV holds genuine predictive utility for depression is both timely and essential. For depression, numerous studies have demonstrated cross-sectional associations between reduced HRV and higher depressive symptoms, or the diagnosis of depression. Far fewer studies have examined whether HRV could be a predictive biomarker to predict the course, treatment response, or remission from depression over time. As a result, despite the growing enthusiasm about measuring HRV, its diagnostic value is based on correlative evidence, and its predictive value remains uncertain.

Furthermore, existing meta-analyses fail to provide an integrative overview limited by their own strengths, e.g., strict inclusion and exclusion criteria. The focus of such work often considers cross-sectional or longitudinal evidence separately from each other, thereby not providing an integrative synthesis of both. In this narrative review, we therefore aim to provide an accessible starting point for researchers who may feel overwhelmed by the breadth of work on heart rate variability (HRV) and depression, by summarizing evidence for and against cross-sectional alterations in HRV in depression, on its predictive value for future depression occurrence and on depression-related interventions (pharmacological and non-pharmacological) and their impact on HRV. We first summarize selected studies and meta-analytic evidence examining HRV both cross-sectionally and longitudinally. We then outline key methodological considerations, including potential confounding factors and practical guidance for designing new studies. Importantly, as this is a narrative review, it is not meant to replace but rather complement existing meta-analyses. We make no claim at completeness as our aim was not to provide an extensive overview, but to provide researchers starting in the field with an introductory overview of studies that might be missed in other literature regarding the topic.

## 2. Methodology

For this narrative review, the authors conducted a literature search using PubMed and Google scholar (adapted terms), with no restriction on the start date and coverage through 30 March 2025. The search strategy employed the following terms (“heart rate variability” OR “HRV” OR “ECG”) AND (“depressi*”) in the title or abstract to identify meta-analyses, and filters were set to identify meta-analysis. For longitudinal studies, the terms (“follow*” OR “longitudinal”) in the title/abstract were added with an AND term in the search string and filters were set for study designs (Adaptive Clinical Trial, Clinical Study, Clinical Trial, Clinical Trial, Phase I, Clinical Trial, Phase II, Multicenter Study, Observational Study). Studies were considered eligible if they met one of the following criteria: (1) meta-analyses examining associations between heart rate variability and depression; (2) cross-sectional studies involving individuals with a diagnosis of depression or clinically relevant depressive symptoms, with a sample size greater than 50 participants and no experimental manipulation (e.g., stress tasks, cognitive challenges, pharmacological interventions or comparable); or (3) longitudinal studies including pre–post assessments. Consistent with the narrative nature of this review, smaller or methodologically diverse studies were also included when deemed informative by the authors, particularly in areas where meta-analytic evidence is lacking, specific age groups are underrepresented, or unique study designs, outcomes, or covariates provide additional conceptual insight. This approach was chosen to synthesize key findings and emerging themes rather than to provide a comprehensive or systematic appraisal of the literature. If two meta-analyses were published within a short timeframe, comprising a majority of similar studies, a consensus was found between both authors on which article to include.

## 3. Innervation of the Heart and HRV

The hypothesis that HRV could reflect the cardiac vagal modulation and resilience to stress, is based on the innervation of the heart by the vagus nerve [10]. The vagus nerve is the 10th cranial nerve that is best known for connecting the brain with the gastro-intestinal system [11] and the heart. It is the key nerve of the parasympathetic branch of the autonomic nervous system [10]. The parasympathetic nervous system is in charge of the ‘rest and digest’ function: it helps the body relax and perform functions, such as digestion, and lowers heart rate (HR) [11]. Its counterpart is the sympathetic nervous system, which is typically activated in stress-related situations or the ‘fight-or-flight’ mode, situations where the body is alert and prepares for action, and situations that coincide with an increased HR [12]. For the body to achieve this balance, the interaction of these two components is crucial [11,12] in order to save energy and mobilize the energy needed for physical action. Our particular interest in HRV can be better understood by looking at the sympathetic and parasympathetic innervation of the heart, organized in a network called the cardiac plexus [13]. The heart receives input both from the right and left vagus nerve (efferent and afferent fibres) and the sympathetic trunk which influences HR, cardiac output, and the force of contraction [13]. The vagus nerve is also connected to the major pacemakers in the heart: the sinus node and atrioventricular node [13]. Through release of acetylcholine, the intrinsic pace of the heart is slowed down (of around 100–110 bpm to a resting heart rate of around 60–80). Conversely, the release of norepinephrine by the sympathetic nervous system increases HR [14]. HRV is defined as the variation in time between consecutive heartbeats, or RR intervals. Given the above described balance between parasympathetic and sympathetic input, higher HRV is generally considered as healthy, reflecting the capacity of an organism to adapt to environmental demands, while low HRV may reflect reduced adaptability to environmental demands [14].

## 4. HRV Measurement and Parameters

HRV is typically measured via electrocardiography (ECG) as the gold standard, or through photoplethysmography (PPG) in most wearable sensors, and is typically recorded as short-term (i.e., typically between 1 and 5 min, in the laboratory setting) or as 24 h recordings (i.e., with wearable technology or Holter monitoring). The most commonly used parameters include frequency-domain measures (e.g., low-frequency [LF] and high-frequency [HF]-HRV and their ratio, reported in Hz) and time-domain measures, such as the root mean square of successive differences (RMSSD reported in ms) as well as HR in beats per minutes. It is beyond the scope of this review to provide an extensive overview of HRV measures, but the interested reader is referred to Shaffer et al. [15] and the most commonly used indices are summarized in Table 1. As mentioned above, in general, higher HRV is thought to reflect greater autonomic flexibility and adaptive capacity, indicating a healthy nervous system response to environmental demands. Understanding these nuances is critical when interpreting HRV in the context of depression- and stress-related disorders. Importantly, HRV metrics are indirect indices of autonomic nervous system activity, rather than direct measures of (cardiac) sympathetic or parasympathetic function. Therefore, while particularly HF-HRV and RMSSD are often used to describe “vagal tone”, this is only partially correct, requiring specific assumptions to be met. These assumptions—and limitations—have recently been summarized in a hallmark paper by Quigley et al. [16]. In brief, to be used as an indirect index of cardiac vagal modulation, recordings need to be made at rest, with minimal postural changes throughout the recording. Importantly, to be used as a proxy for cardiac vagal modulation, respiration must fall within the typical adult frequency range (approximately 7–24 breaths a minute) and respiration should be explicitly measured or controlled. Furthermore, to be reliable, only high-quality ECG recordings should be used. Provided that the phasic vagal modulation scales with the tonic parasympathetic activity, HF-HRV may serve as a reasonable proxy for individual differences or condition-related changes in cardiac vagal control, though it should not be interpreted as a direct measure of absolute vagal tone; see Quigley et al. [16]. In contrast, LF and the LF/HF ratio, which were previously used to assess the sympathetic modulation and sympatho-vagal balance, have been criticized and are not thought to reliably measure this activity, due to the LF-frequency band largely reflecting baroreflex activity and likely having mixed sympathetic–parasympathetic input. The scope of this review is therefore to focus mainly on measures reflecting cardiac vagal modulation. We provide an overview of studies and the HRV parameters investigated, and report estimated effect sizes as they were reported in the meta-analyses (standardized mean difference (SMD), Cohen’s d or bias-corrected Hedges’ g, particularly suitable for smaller studies).

## 5. HRV and Depression

### 5.1. Cross-Sectional Studies

#### 5.1.1. HRV and Depression in Adults

The most consistent evidence for an association between diagnosed depression and heart rate variability (HRV) arises from resting-state measures in cross-sectional studies in adults, particularly those studies approximating cardiac vagal activity, such as high-frequency HRV (HF-HRV) and the root mean square of successive differences (RMSSD), which show relatively consistent associations in adult populations. Among the earliest and most comprehensive studies investigating HRV and depression is the Netherlands Study of Depression and Anxiety (NESDA): In a sample of 2373 individuals, including 524 controls, 774 participants with remitted MDD, and 1075 with current MDD, both remitted and current MDD groups exhibited lower mean SDNN and respiratory sinus arrhythmia (RSA) compared with controls [17]. However, most of this difference was explained by antidepressant medication intake; after adjusting for medication use, SDNN was no longer significant and the effect for RSA was substantially reduced, raising the question of whether this reduction is a marker of antidepressant intake rather than depression per se (see also [17] and section on confounding factors below). However, some studies also find an association in unmedicated subjects, such as the meta-analysis by Kemp et al. [18], who found reduced HF-HRV, increased LF/HF-ratio, and a negative association between depression severity and overall HRV (r = −0.364). Importantly in this analysis, only patients with a diagnosis of MDD, but without current antidepressant medication were included. Other meta-analyses of HRV in unmedicated patients with a more granular analysis have since also reported small, but significant effect sizes for reduced HF-HRV (e.g., g = −0.32) and small-to-medium effect sizes for reduced RMSSD (e.g., g = −0.46) [19]. The latter analysis included 2250 non-medicated patients with depression and 1982 healthy controls. Next to RMSSD and HF-HRV, they also showed significant differences between patients and controls for frequency and time domain measures including LF-HRV, LF/HF-HRV ratio, VLF-HRV, IBI, and SDNN. Similar findings with a slightly larger effect size were observed when antidepressant status was not considered an exclusion criteria, with a slightly higher sample size, e.g., in the meta-analysis of Wu et al. [20], with 2359 participants with depression and 3547 controls (RMSSD: g  = −0.51, HF-HRV: g  =  −0.51). Similarly to Koch et al. [19], these authors also showed differences in HF-HRV, LF-HRV, RMSSD, and SDNN, and additionally, a reduction in PNN50. Besides the association of diagnosis and depressive symptomatology, it is also of interest to assess the association between depressive symptom severity and potential reductions in HRV. In other words, is there a dose-dependent relationship between HRV and depression severity? To date, few meta-analyses focus on this relationship in adults, but the data by Kemp et al. [18] seem promising.

#### 5.1.2. Meta-Analyses in Children and Adolescents

Cross-sectional associations between depressive symptoms and HRV have also been reported in younger populations. Koenig et al. [21] performed a meta-analysis on case–control comparison (four studies), as well as depressive symptoms, as a continuous measure (six studies) in children and adolescents. A reduction in HF-HRV was seen in the case–control design, but no association with depression severity was detected in the sample with continuous measures. This was later confirmed in an updated analysis [22], which included more studies: again the group difference was significant, but association with depressive symptoms was not. Chen et al. [23] also investigated HRV in adolescents and children in a smaller sample of around 400 controls and 400 people with MDD. Similarly to the meta-analyses focusing on adults, a reduced HF-HRV (g =  −0.38), RMSSD (g =  −0.49), and PNN50 (g = −0.79) was found in individuals with depression, compared to healthy controls. In this analysis, LF-HRV and SDNN did not differ between groups. This is in line with the most recent meta-analysis in children and adolescents [24], which included 31 articles, involving 4534 children and adolescents. They confirmed a negative association between depression severity and HF-HRV, with a moderating effect of age, and RMSSD, but not for LF-HRV or SDNN. The fact that HRV is lower in children with depressive symptoms suggests that the observed HRV alterations are not merely a consequence of long-term sedentary behaviour or chronic illness burden.

#### 5.1.3. Data on HRV and Depression in Older Populations

By contrast, Brown et al. [25] analyzed HRV in a depressed population with a mean age older than 60 years, both in clinical and community settings. Their research showed that HRV was reduced among older adults with clinical depression (*N* = 550), relative to healthy controls (*g* = −0.334, *p* = 0.007). Contrary to the other populations, only LF-HRV was significantly reduced in depressed patients (*g* = −0.626, *p* = 0.007). HF-HRV, while reduced, was not significantly lower in the MDD compared to the HC group (*g* = −0.331, *p* > 0.05).

In conclusion, most population-based studies and meta-analytic effect sizes support the finding of small-to-moderate reductions in HRV. It is however important to appreciate that factors such as medication affecting the cardiovascular system, antidepressant medication intake, age, sex, and social context can influence these findings. Furthermore, a methodological limitation of the meta-analytic studies above is the high overlap of studies that were included in the meta-analyses. Additionally, for all of the meta-analyses, measures of heterogeneity (*I*^2^) are generally very high, possibly indicating various subgroups of depression and influential covariates, which we will discuss further below. An overview of all meta-analytic evidence can be found in Table 2.

### 5.2. Longitudinal Observational Studies for Depression and HRV

Table 3 shows an overview of all longitudinal/interventional studies. Longitudinal evidence assessing HRV as a potential predictive biomarker for depression is still limited compared with cross-sectional research. One of the largest longitudinal dataset to date, the *Whitehall II study* [26], followed over 2200 participants for an average of 10.5 years. Lower baseline heart rate and higher HF-HRV and RMSSD were associated with a reduced likelihood of developing depressive symptoms at follow-up in male participants without baseline depression (in females, this relationship was not significant, albeit the female sample was much smaller). Notably, baseline depressive symptoms did not predict HRV at follow-up, suggesting a possible unidirectional relationship.

Interestingly, in a smaller, more recent study, 146 male twins were followed for seven years, and a similar association was found: baseline HRV was robustly and negatively associated with Beck Depression Inventory (BDI) scores, and this association was not explained by either demographics, nor antidepressant use [28]. Like the *Whitehall II* study, the authors also tested the temporal associations between baseline BDI scores and HRV and found that while higher BDI scores did predict lower HRV at follow-up, this effect was explained by antidepressant use. This may suggest that while baseline HRV could be useful to predict depressive symptoms, the inverse is not true and may be linked to antidepressant usage. In line, in two smaller studies, in adolescents and college students, baseline HRV predicted depressive symptoms one year later [29,44].

While associations between HRV and depressive symptoms are reported, contrary findings have also been reported in older populations. An et al. [30] conducted a five-year community-based study in adults aged 65 years and older and found no association between baseline HRV and depression scores. Instead, higher HF-HRV at baseline was unexpectedly linked to an *increased* risk of developing depression at follow-up, suggesting that age is an important factor to take into account (see Section 6 on confounders and factors influencing HRV). Beyond depressive symptoms, the above-mentioned Whitehall II study, using 5936 participants [26,27], also found that participants with both high depressive symptoms and a resting heart rate of 80 or above at baseline had a significant, three-fold higher risk of all-cause mortality compared to those with lower depressive symptoms and resting heart rates between 60 and 80 bpm, when considering a follow-up time of ~5 years, emphasizing the importance of assessing and addressing symptoms transdiagnostically [27].

Together, these longitudinal observational findings extend beyond cross-sectional associations by clarifying the directionality of effects between autonomic regulation and depressive symptoms. The observation that baseline HF-HRV predicts subsequent depressive symptoms, but not vice versa, suggests that it may be a vulnerability marker rather than a transient state or trait marker of depression. Specifically, lower baseline HF-HRV may represent ongoing limitations in autonomic flexibility and regulatory capacity that predispose individuals (particularly males) to later increases in depressive symptoms. Within the neurovisceral integration model, this pattern is theoretically meaningful: reduced HRV would reflect weakened top-down prefrontal modulation of limbic and brainstem systems involved in emotion and stress regulation. Consequently, compromised autonomic–neural coordination may be a mechanistic pathway through which the long-term risk for depressive symptomatology is increased over time.

### 5.3. Longitudinal Studies Using Antidepressant Treatment

Most other longitudinal studies have investigated the relationship between HRV and depression after treatment. In their meta-analysis, Kemp et al. [18] summarized studies published before 2010, including 637 patients with depression, and found that while HRV is reduced in depression (see above) in the meta-analysis of 186 patients with pre–post data, treatment with antidepressant medication (except TCAs) did not change overall HRV. Since then, several studies have been published on the topic.

For instance, Hartmann et al. [31] examined 62 antidepressant-free MDD patients and 65 healthy controls and did observe HRV differences at baseline, in particular for HF-HRV power (eta2 = 0.115,) RMSSD (eta2 = 0.038), and LF-power (eta2 = 0.04). Here, the difference between groups (control or depression diagnosis) was significant for HF and LF power, but not RMSSD. In continuous analysis, HRV parameters did not predict depression severity. Interestingly, after 2 weeks of antidepressant treatment, depression severity was reduced significantly, and the change scores correlated with changes in HF-HRV power, indicating that increasing HF-HRV power correlated with a reduction in depression severity [31]. Further insights come from other systematically conducted treatment studies. One of the largest studies focusing on HRV [32] investigated the association of baseline and change in HRV with depressive symptoms after treatment using transcranial direct current stimulation (tDCS) or sertraline. In their sample of 116 patients with MDD who participated in the double-blind randomized controlled trial, baseline values were lower in the MDD group (RMSSD, HF-HRV) and did not change after treatment with AD medication or stimulation, nor was it useful to predict response. Another study examined 66 patients treated with escitalopram or quetiapine fumarate extended release for 12 weeks and found that while HRV measures did not change during treatment, baseline RSA and LF-HRV were higher in responders [33]. Interestingly, in an early study with 16 subjects, it was found that stratification using RSA was useful in predicting response: those with little change in RSA from before to after treatment showed lower reductions in depression severity than those with more change in RSA. Stratification by change in RSA may thus help to identify a biological subgroup [34]: it may be possible that RSA change reflects a biological “responsiveness” phenotype (e.g., preserved capacity for adaptive autonomic regulation) rather than a depression subtype per se. If validated, RSA trajectories might contribute to risk stratification or early treatment monitoring, complementing symptom-based assessments. These hypotheses remain exploratory in the context of the small samples and warrant confirmation in adequately powered studies.

### 5.4. Non-Pharmacological Interventions and HRV

Regarding non-pharmacological interventions for HRV, several interventions have been discussed, but studies are still rather limited. A recent review and meta-analysis summarizes all available studies encompassing non-pharmacological interventions and their impact on HRV (including exercise, biofeedback, and psychological interventions, albeit also in non-clinical/non-depressed participants). Importantly, they found no significant effect for psychological interventions nor for biofeedback, but showed improved HF-HRV and RMSSD with yoga/tai chi [45]. To add to these findings, for studies strictly focusing on depression, Neyer et al. [35] followed 50 patients with depression during standard care (psychotherapy with the majority on antidepressant treatment). While clinician-rated depressive symptoms correlated with HF-HRV and RMSSD at baseline (r = −0.360 and r = −0.352, respectively), they were no longer correlated at the follow-up, and while there was an improvement in depressive symptoms, no change was detected for either RMSSD or HF-HRV. Similarly, studying 30 patients with depression who had a diagnosis of stable coronary heart disease and received cognitive behavioural therapy (CBT) for up to 16 sessions, no improvement for HRV was found in patients with mild or moderate depression; however, in patients with severe depression, RMSSD normalized to control level [36]. In the third study, following 124 patients with coronary heart disease and depression who received up to 12 sessions of CBT, results indicated that before treatment, non-remitters showed significantly higher nighttime heart rates and lower nighttime VLF-HRV compared to remitters, even after controlling for potential confounding factors [37]. More recent trials add nuance to the mixed CBT findings. In an antidepressant-free sample, Euteneuer et al. [38] randomized 80 patients with MDD to 14 weeks of CBT vs. waitlist and assessed 24 h ECG. They reported an increase in overall (global) HRV, but no significant changes in HF-HRV or LF-HRV. Notably, effects appeared moderated by baseline symptom severity, such that participants with higher self-rated depression (BDI-II > 34) showed greater improvements in daytime HRV indices (including daytime HF-HRV and LF-HRV) from baseline to post treatment compared to waitlist. Another shorter multimodal CBT-based programme incorporating breathing relaxation (12 sessions over 4 weeks) in patients with MDD produced modest, partly time-limited increases in HF-HRV and group differences in LF-HRV/LF-HF, but effects were small in absolute magnitude and the sample was predominantly male [40]. Beyond CBT, a 9-month cluster RCT in antidepressant-free older adults with subthreshold depression found that behavioural activation (12 weekly sessions plus homework) led to greater improvements than usual care in SDNN and lnHF (with no significant change in LF/HF), although the sample remained relatively small and included subthreshold rather than clinically diagnosed depression [39].

Finally, newer studies have investigated the effect of biofeedback. Intervention studies using HRV biofeedback have demonstrated that HRV can be effectively increased through targeted training. A recent meta-analysis has shown that HRV biofeedback led both to a reduction in depressive symptoms and an increase in vagally mediated HRV in adults with various disorders [41]. Through the autonomous nervous system, HRV and depression share pathways that can be modulated by adjusting breathing frequency as an example of a voluntary action. This relationship between HRV and ‘malleable’ vegetative functions, such as breathing, makes it a good candidate for biofeedback, as patients can adapt to paced breathing and other interventions. Biofeedback as an intervention enhances self-efficacy and interoception, which also affects other systems related to stress and resilience, including HRV. Studies revealed that HRV biofeedback effectuates acute improvements during biofeedback practice [46]. However, long-term results and the impact on clinical outcomes remain unclear [46]. For MDD, results are mixed: though HRV changes over time are inconclusive, biofeedback with focus on HRV seems to positively affect depression symptom improvement [46]. The baroreflex system, which causes large oscillations in heart rate when breathing at a particular frequency, has been suggested as a mechanism through which controlling breathing can positively influence heart rhythm and HRV [47]. One small, first randomized controlled study using biofeedback in 20 patients with depression found that adding HRV biofeedback to psychotherapy yielded greater increases in SDNN and greater decreases in depressive symptoms in patients with MDD compared to treatment as usual, with no change in HF-HRV or LF-HRV. Interestingly, the improvement in depressive symptoms was partially mediated by improvements in HRV (SDNN) [42]. Another study showed that in 48 patients with MDD [43], after 6 weeks of 60 min weekly biofeedback training sessions, the HRV-biofeedback group had lower breathing rates and higher HRV indices (SDNN, ln [LF], and LF/HF ratio) at post testing, as compared to the control group. Pre–post levels of depressive symptoms also decreased significantly. These findings strengthen the argument for a meaningful association between HRV and depression. However, current evidence does not yet allow us to draw definitive conclusions and more studies on HRV biofeedback, in clinically depressed patients, are needed.

In summary, evidence linking treatment to HRV changes in depression remains mixed, and patterns differ by intervention type. In pharmacological RCTs (as gold standard), samples are typically more homogeneous and follow-ups are relatively short; many studies show clear symptom improvement but no or only small average changes in HRV, and associations with outcome are inconsistent, sometimes appearing only in correlational change-score analyses or responder/non-responder contrasts rather than at the whole-group level. In contrast, large naturalistic cohorts such as NESDA include a broader, more clinically representative population and longer follow-up, and they suggest an overall HRV reduction over time that is linked to antidepressant use. A straightforward way to reconcile these findings is that symptom improvement may be accompanied by increases in HRV in some individuals, while antidepressant exposure may be associated with reductions in HRV, so the average net effect is small, inconsistent, or even appears null, especially in shorter trials that mix responders and non-responders and are not powered for physiological outcomes. If this explanation fully accounted for the pattern, a clearer HRV increase would be expected in purely psychological interventions. However, the (still relatively small) CBT literature largely shows symptom improvement with little average HRV change, except in specific subgroups (e.g., more severe depression)*,* indicating that this explanation is insufficient to explain the absence of change. In contrast, HRV biofeedback studies report increases in HRV indices alongside symptom reductions more consistently. This implies that the HRV–symptom coupling is not uniform and may depend on the intervention’s targeted mechanisms and/or on patient subgroups. More broadly, depression treatment studies often show sizeable symptom reductions even in control/placebo conditions due to non-specific influences (e.g., spontaneous remission/regression to the mean, expectancy and therapeutic contact, improved sleep/activity from study participation, and changes in concomitant treatment), further complicating detection of HRV-specific effects. Future studies should therefore carefully model medication exposure and symptom trajectories, and prospectively test stratification approaches (e.g., using low baseline HF-HRV or related profiles) to identify subgroups in whom HRV is more tightly coupled to clinical change.

## 6. Individual Predisposition and Factors That Influence HRV

The above studies have made it clear that important confounding factors influence the associations between HRV and MDD. In the following section, we will take a closer look at such factors including genetic predisposition, demographic factors, and study-related factors.

### 6.1. Genetics and HRV

Heart rate is a polygenic trait, which means that multiple genetic variants contribute to the personalized characteristics and ‘genetic make-up’ of HRV for each individual [48]. Heritability is estimated to range between 14 and 71% [49]. The single-nucleotide polymorphism (SNP)-based heritability, based solely on genetic information, is estimated to be 9.45% for RMSSD and 7.86% for SDNN [50]. Candidate-gene studies have found indications that the genetic variation in pathways related to the brain-derived neurotrophic factor (BDNF) and serotonin is linked to HRV phenotypes, particularly also in the context of anxiety [51]. A meta-analysis of genome-wide association studies (GWAS) primarily interested in the link between genetics and HRV as a trait showed 493 independent genetic variants in 352 genetic loci. Genes that contribute to genetic aspects of resting heart rate could be traced back to cardiac biology, including cardiac tissue development, muscle cell differentiation, and pro-arrhythmogenic pathways [48]. The genetic correlation between RMSSD and SDNN was 98.4%. Based on genetic correlations, further evidence was found for shared genetic effects of RMSSD with diastolic blood pressure, systolic blood pressure, heart rate, heart failure, coronary artery disease, and type 2 diabetes [49]. Based on these genomic estimates, lower HRV measures were associated with increased all-cause mortality, though independent of the underlying genetics [49]. The observation that this relationship is not mediated by one’s genetic predisposition suggests a high involvement of environmental factors, e.g., chronic stress, in the relationship between HRV and all-cause mortality [49], as chronic stress is a shared risk factor for both reduced HRV measures and all-cause mortality. In-depth research looking into shared genetic vulnerability seems more focused on anxiety than depression; however, as psychiatric phenotypes, anxiety and depression are strongly related and often comorbid. Further investigating gene–environment interactions and the interaction of genetic predisposition for HRV in combination with particular environmental stressors could be a valuable next step in understanding the role of HRV genetics and its potential as a biomarker for depression subtypes. However, for now, this relationship has not been investigated to the extent of differentiating between phenotypic subtypes or depression trajectories. More research is needed for this gene–environment interplay to be extrapolated towards patient stratification.

### 6.2. Sex-Dependent Effects on HRV

Throughout the literature, sex and/or gender differences are often used as covariates, but analyses are often not presented in a stratified fashion. Tegene et al. [52] published reference values for HRV and found subtle differences in men and women with longer interbeat interval (IBIs) in men.

In a meta-analysis of 63,612 participants (31,970 females), it was found that although women have a higher heart rate and lower overall HRV than men, they show relatively greater parasympathetic (vagal) influence reflected by higher HF-HRV power, together with lower LF-HRV and V-LF, while men show greater sympathetic dominance [53]. Another meta-analysis discussing sex-related differences in diurnal variation in the time domain indices of HRV showed that males had significantly longer RR intervals (SMD = 0.57, *p* < 0 .001, 2711 subjects, 21 studies), a higher SDNN (SMD = 0.56, *p* < 0.001, 2538 subjects, 18 studies) and a higher RMSSD (SMD = 0.18, *p*  < 0 .001, 1531 subjects, 14 studies) compared to females [54]. In the frequency domain analyses, men showed significantly higher TP (SMD = 0.50, *p* < 0.001, 692 subjects, six studies) and LF/HF ratio (SMD = 0.59, *p* < 0.001, 1641 subjects, 10 studies) compared to women. Differences were more pronounced at night [54]. Furthermore, sex hormones, in particular oestrogens, may account for some of the observed phenomena in HRV through peripheral effects or direct effects on the brain [55], resulting in heterogeneity also within the female population. Overall, a decrease in vagal dominance on the heart is assumed to occur from the follicular to the luteal phase with higher LF power and LF/HF ratio towards the luteal phase and HF power decreasing from the follicular to the luteal phase [56]. It should be noted that many of the older articles used “sex” and “gender” inappropriately, often referring to biological sex as gender. We would here like to highlight that it is crucial to assess both the effects of sex and gender in future studies, since gendered social and cultural factors likely influence HRV. Differential exposure to chronic stressors (e.g., caregiving burden, workplace harassment, minority stress) may lower resting HRV [57] and increase depressive symptoms [58], and may thus be a moderator of associations between depressive symptoms and HRV. Gender norms may also influence coping styles, including emotion suppression or rumination. Finally, gendered patterns in healthcare-seeking and diagnosis can bias the grouping of ‘depression’ toward different severity, chronicity, or comorbidity profiles that are themselves linked to HRV, thereby moderating or distorting associations independent of biology [59]. Unfortunately, since most studies use the term gender incorrectly (i.e., actually referring to sex), this often leads to wrong conclusions on the effects of gender and further dedicated research on the effects of gender is needed. 

### 6.3. Age and HRV

It is well-known that age has a pivotal effect on HRV. This is well illustrated by the reference values published by [52] showing that RMSSD decreased steadily from adolescence to old age with younger people having an RMSSD mean well above 50 ms, steadily dropping in older people with values of less than 28 ms for those 65 or above. Interestingly, some have suggested a turning point after 70 years of age, with small increases observed in populations above 70. This is highly relevant for analyses in the elderly and may provide explanations for findings such as reported by An et al. [30]. In a study using data from more than 8 million individuals using PPG-derived HRV, it was shown that while all HRV indices decline with age, vagally mediated HRV indices declined faster [60]. It was also reported that for SDRR, RMSSDRR, and pNN50, there is a positive correlation with age until the age of 12 years. After that, a negative correlation was observed in healthy individuals with ages until 74 years of age [61]. The authors also discuss a stronger involvement of sex differences above the age of 12 [61]; it can be assumed that this coincides with the onset of puberty and the increasing role of sex hormones, as discussed above. Calderón–Juárez et al. discussed that age and HRV relate in a non-linear manner (also above the age of 12) [62]. They included 1121 subjects between 18 and 92 years of age and observed that groups older than 30 years of age show smaller values of SDNN and RMSSD compared to the 18–29 years group (same sex). Here, few differences were observed between males and females in all linear HRV indices (same age group).

Taken together, these findings indicate that age and HRV associations should not be assumed to be linear across adulthood, and that the age range of ~70+ may represent an important distinction from previously observed age-related declines. Therefore, it is advisable to perform age-stratified interpretation and/or explicit non-linear modelling of age (e.g., splines or age–band interactions), particularly in cohorts enriched for adults over 70 years. This is directly relevant to interpreting the seemingly contradictory associations discussed above: differences in cohort age and the inclusion of very old participants can plausibly shift the observed direction and magnitude of associations, providing a confounding explanation for findings such as those reported by An et al. [30]. Accordingly, comparisons across studies should explicitly test and report age distributions and consider separate estimates for older age groups where feasible.

### 6.4. Lifestyle and Physical Fitness

Lifestyle aspects, such as physical activity, body mass index (BMI), and smoking have also been reported to contribute to variability in HRV [7] and are modifiable lifestyle factors. In the above study of 8 million individuals [60], it was shown that increased physical activity (as measured with step count) was correlated to increased HRV in a dose-dependent manner. It is also known that metabolic regulation (long-term) is a strong modulator of HRV, but the contribution of BMI may not be linear [62]. For instance, in a recent, large-scale study across 3159 patients with psychiatric disorders (schizophrenia, mood, or anxiety disorders), it was shown that next to differences within the disorders, HRV for the people with BMIs in the underweight and normal weight categories was higher than for those in the overweight category [63]. Another study examining 34 obese individuals and an interventional paradigm (Trier Social Stress Task) showed that obese individuals had a lower stress reactivity for HR, and less recovery in RMSSD following the stress task, compared to healthy weight controls [64]. Regarding physical activity and metabolic differences, a meta-analysis of HRV and exercise in individuals with diabetes showed an increase in SDNN (estimated effect size = 0.59), RMSSD (estimated effect size = 0.62), pNN50 (estimated effect size = 0.62), HF-HRV (estimated effect size = 0.58), and a decrease in LF-HRV (estimated effect size = −0.37) and LF/HF ratio (estimated effect size = −0.52) [65]. A systematic review focusing on individuals older than 65 years showed a consistent positive effect of endurance exercise on multiple HRV parameters. Within-group or between-group analysis demonstrated significant improvements following training interventions for HF and RMSSD, SDNN, and LF/HF ratio. Similar effects were discussed for multimodal training for RMSSD, HF, and mRR. Significant decreases were found in RHR, LF nu, and LF/HF [66]. For smoking and alcohol, results are somewhat ambiguous, depending on the evaluation of acute consumption effects, long-term effects, and for smoking time after cessation [67].

These lifestyle factors have been linked to depression and depression severity, independent from HRV. BMI is a covariate often taken into account for depression studies (including physical activity as a confounding variable), but is also becoming increasingly popular for depression research as part of an intervention [68]. Future research on HRV and depression should consider thoroughly assessing physical exercise, as well as smoking and drinking habits, including them in the analyses to increase understanding of HRV in the context of depression and the observed heterogeneity between studies. Furthermore, lifestyle interventions (e.g., regular aerobic activity, sleep regularization, paced-breathing/HRV biofeedback) targeting BMI normalization and/or smoking cessation/healthier habits may complement antidepressants by targeting physiological regulation more directly, potentially offsetting medication-associated HRV reductions while supporting symptom improvement. Clinically, they may prove useful as add-ons for partial responders and for longer-term recovery/relapse prevention, with additional cardiometabolic benefits.

### 6.5. Antidepressant Medication Intake

As briefly mentioned above, it is well-known that antidepressant medication can decrease HRV. In the previously mentioned NESDA study [17], it was shown that antidepressant medication (tricyclic antidepressant medication, but also SSRIs/SNRIs) reduced the association between depression and RMSSD significantly. The longitudinal analysis with a focus on antidepressant medication showed that the negative association between RSA and antidepressant medication was sustained over a 2-year follow-up period. These findings are also consistent with cross-sectional associations from the Irish Longitudinal Study on Ageing (TILDA), which found that in 317 elderly participants with depression, who were not taking antidepressants, HRV did not differ from controls on any measures of HRV, but all antidepressants were associated with lower measures of HRV [69]. Unfortunately, depression severity was not controlled for in this analysis [17,70]. There is an obvious discrepancy between the longitudinal, naturalistic studies in larger samples finding negative effects (i.e., antidepressant medication explained most of the effects on HRV) and the meta-analyses in unmedicated patients [18,19], who do find significant reductions in HRV in depression, even in the absence of medication. This discrepancy could reflect differences in depression subtype or comorbidities and other factors differing between unmedicated meta-analytic samples and naturalistic cohorts. Additional explanations include time-varying medication effects (i.e., when was treatment initiated/titration of the medication), or could reflect response-dependent effects, where medication-related HRV reductions may mix with recovery-related increases in HRV for responders. It is also possible that naturalistic samples capture more complex and cumulative antidepressant exposure (e.g., prior antidepressant failures, switching/augmentation, recent discontinuation) and polypharmacy, which may add to or prolong HRV reductions. Therefore, future studies should take care to include detailed lifetime/recent medication histories (dose, duration, switching) and explicit polypharmacy indicators. Ideally, HRV changes would be measured in the context of randomized controlled clinical trials to examine HRV trajectories around medication changes in a controlled environment. Finally, an argument often made is that patients receiving antidepressant medication may suffer from more severe depression than those without antidepressant medication; particularly in light of some studies reporting a correlation of depressive symptoms and HRV, this factor should be considered.

## 7. Methodological Considerations and Confounding Factors: Designing Your Own Study

From the overview discussed in the manuscript, we list the most important aspects to acknowledge when designing your own study in patients with depression and attempt to make a list of essential versus recommended conditions. Importantly, these strongly depend on your research question and should not be taken over as is but rather be used to consider important covariates. More general guidelines on factors to considered for HRV can be found elsewhere [15,16].

### 7.1. Participant Characteristics to Assess

#### 7.1.1. Essential

For studies in participants with depression, it is mandatory to carefully collect participant characteristics known to influence HRV, and which may moderate or mediate the association with depression. These include age, sex, BMI, antidepressant medication intake (at least current medication and dosage, and whether polypharmacy is present), as well as any medication influencing the cardiovascular system (e.g., particularly beta-blockers, and other antihypertensives, stimulants, benzodiazepines, antipsychotics, and thyroid medication) and, depending on your study design, exclude or document cardiac disease.

#### 7.1.2. Recommended

In addition, it is advisable to include the following: menstrual phase (and contraception intake) either as a controlled variable or document it, gender, and (psychiatric) comorbidities, but also sample characteristics generally associated with a lower HRV in psychiatric populations, including chronic and current stress, depression severity and anxiety, as well as smoking status (or, if smoking is restricted, to document this carefully). Finally, it is useful to include a measure of physical fitness.

### 7.2. Environmental Controls

#### 7.2.1. Essential

In a laboratory environment, it is necessary to keep room temperature stable and noise to a minimum in order to reduce bias; furthermore, exercise should not be allowed on the day of testing (also consider participants cycling to the appointment) and ideally, no strenuous exercise should be permitted on the day before the appointment. Also consider that if research facilities are to be reached by stairs, an elevator should be used if possible and an acclimatization phase should be considered to control for breathing and other physiological parameters linked to physical activation, allowing these to normalize before starting the investigation. Furthermore, caffeine consumption and smoking should be restricted before testing, depending on your research question (but at least in the 1–2 h prior to recording, constant across participants).

#### 7.2.2. Recommended Controls 

Where feasible, it can further reduce heterogeneity to standardize and report how acclimation is implemented (e.g., seated posture and minimizing talking or other distractions), especially when comparing across sessions or cohorts; alcohol consumption on the day prior to the testing should be limited. Finally, food intake should be kept stable (i.e., not test sober people together with those who just had lunch).

### 7.3. Measurement Protocols

#### 7.3.1. Essential Controls

Measurement protocols can induce heterogeneity, which can be minimized and controlled through good preparation and strict application of the protocol. Given the important diurnal variations in HRV (e.g., increase in RMSSD in the night until morning, then decrease), if HRV measures are performed in the morning, it is essential to consider sleeping patterns and time since waking up, as well as the time of administration. This becomes particularly important for repeat measures, where it is essential to keep the time of the second assessment identical. Ideally, all participants should be tested at the same time of day, preferably in the afternoon (also relevant if also cortisol measures are taken).

#### 7.3.2. Recommended Controls 

Because HRV measurements largely rely on a normal breathing rhythm, breathing patterns should be considered or measured, if possible, particularly if there is interest in HF-HRV.

### 7.4. Considerations for Studies Using Wearables

As wearables take over and contribute significantly to research on physiological measures such as HRV in an everyday context, it is necessary to be aware of the advantages and limitations of these technologies. To our knowledge, no consensus exists on the preprocessing requirements, but an overview of commonly used methods can be found here [71]. In our experience, when using wearable-derived data (PPG), phases of physical activity should be clearly separated from sedentary phases, because HRV can be biassed by motion-related artefacts and changes in ambient light [71], leading to reduced signal quality. For HF-HRV, ECG-based standards generally assume clearly identifiable R-peaks and recommend stationary 5 min segments with rigorous beat editing/artefact handling, often complemented with manual control of said artefacts; however, these procedures are not always directly transferable to PPG because the pulse waveform is more motion-sensitive and wearables are typically recorded over extended periods (e.g., 24 h or longer), making manual artefact correction extremely time-consuming. If wearables are used for short-term recordings (i.e., 5 min segments), caution is warranted to ensure participants are correctly instructed regarding the recording situation (like laboratory conditions as described above), and recordings with a high number of invalid IBIs (e.g., >5% of total beats) should be treated with caution. Finally, due to the large inter-individual variation, the main strength of wearable HRV measurement lies in within-subject settings (i.e., change from baseline; longitudinal/interventional designs), permitting control for important covariates, rather than between-subject comparisons without stringent standardization; this may provide a valuable and well-acceptable method to obtain data from patients with depression.

#### Recommended for Wearable Measurements (PPG)

Although wrist-based monitors are commonly used, finger-based methods (i.e., rings) may provide a better PPG signal [72]. We have had good experiences focusing on sedentary periods such as sleep or resting episodes, which can be detected by most modern sensors (e.g., via accelerometry), and using automated filtering in the Kubios HRV scientific software (version released 2023, beat correction low to medium), which produced results that compared well with manual spot checks (unpublished data). Importantly, when removing motion artefacts, it is advisable to record several days of data if circadian rhythm is relevant, to avoid large phases of missing data.

## 8. Limitations

While we attempted to give a good overview of the field, our approach was not systematic, and it is possible that important studies were overlooked. Because this narrative review integrates heterogeneous study designs, we did not conduct a formal risk-of-bias or quality assessment across all included studies, and the strength of evidence should not be interpreted as equivalent across designs. The readers are advised to carefully consider study limitations when comparing the different study designs.

## 9. Conclusions

Taken together, current evidence supports a cross-sectional association between depression and reduced HRV, especially in vagally mediated components, and some evidence suggests that longitudinally, low HRV may predict future depressive symptoms, but not vice versa. However, antidepressant medication use, methodological heterogeneity, and population differences complicate interpretation, with studies often underpowered to properly account for covariates. This challenge is not unique to HRV; it reflects a broader issue within the field of biological psychiatry, where many proposed biomarkers for depression- and stress-related disorders show strong sensitivity but weak specificity (e.g., HRV has also been considered a “biomarker” for anxiety disorders and post-traumatic stress disorder, potentially reflecting autonomic dysregulation or stress vulnerability across disorders rather than a depression-specific biomarker). Longitudinal and interventional data suggest that HRV may hold promise as a biomarker for future depression risk and recovery, although causality remains unresolved. Future studies with wearable technology could help in achieving the sample sizes needed to account for the confounding factors. To move beyond correlational findings, several research priorities follow from our synthesis: (1) adequately powered, preregistered longitudinal studies that repeatedly assess HRV and depression severity while rigorously controlling for key confounders (e.g., antidepressant use/duration, physical activity/fitness, age, sex, BMI and smoking behaviour, comorbid disorders, and cardiovascular/metabolic factors), and that explicitly model within-person change; (2) studies testing whether HRV predicts treatment response and relapse risk across modalities (pharmacotherapy, psychotherapy, exercise), ideally incorporating HRV as a prospective stratification variable rather than only an outcome; and (3) validation of wearable-derived HRV against the gold-standard ECG in clinical samples with depression. Progress on these priorities would clarify temporal ordering, strengthen causal inference, and determine whether HRV can be translated into a robust, clinically meaningful tool rather than a correlate of depression.

## Figures and Tables

**Table 1:** (**a**)**.** Time domain-related HRV measurements. Description of most important parameters describing HRV. (**b**) Frequency domain-related HRV measurements: description of most important parameters describing HRV.

(**a**) Time domain-related HRV measurements	
**Measure**	**Description and Typical Interpretation**
SDNN	Standard deviation of all normal-to-normal (NN) intervals, also known as RR interval (i.e., peak of heartbeat intervals); reflects overall HRV. Higher = better autonomic balance.
IBI	Interbeat interval is the time period between successive heartbeats.
RMSSD	Square root of mean squared differences between successive NN intervals reflects parasympathetic (vagal) activity.
pNN50	Percentage of successive NN intervals with >50 ms difference: parasympathetic activity marker.
SDANN	SD of 5 min segment averages of NN intervals over 24 h; represents long-term HRV fluctuations.
SDNN Index	Averaged variability in 5 min windows represents short-term HRV fluctuations.
(**b**) Frequency domain-related HRV measurements	
**Measure**	**Description and Typical Interpretation**
LF: Low Frequency	Power in low-frequency range (0.04–0.15 Hz) can reflect both sympathetic and parasympathetic activity.
HF: High Frequency	Power in high-frequency range (0.15–0.40 Hz) can reflect parasympathetic (vagal) activity under specific conditions; in particular if linked to typical breathing patterns, see also respiratory sinus arrhythmia.
VLF: Very Low Frequency	Power in very-low-frequency range (0.003–0.04 Hz).
LF/HF: Ratio of LF to HF power	Balance between LF and HF components. It was previously thought of as an autonomic balance; interpretation has been criticized among others due to influence of both PNS and SNS for LF-HRV
TP: Total Power	Total variance in all frequency bands reflects overall HRV.

Abbreviations: SDNN: standard deviation of NN intervals; RMSSD: root mean square of successive differences; pNN50: percentage of NN intervals differing by >50 ms; SDANN: standard deviation of average NN intervals; SDNN index mean of 5 min SDNN values.

**Table 2:** Summary of meta-analytic studies investigating HRV differences in depression.

Authors	Design/Population	N Included Studies and N Cases/N Ctrls		Antidepressant Medication	HF-HRV Compared to Ctrls	RMSSD Compared to Ctrls	LF-HRV Compared to Ctrls	LF-HRV Compared to Ctrls	LF/HF Ratio Compared to Ctrl
**Kemp et al. (2010; [18])** *1	Adults without current antidepressant medication	18	673/407	none (excluded)	Lower HF-HRV (g = −0.210, *p* < 0.027, *I*^2^ = 0.28)	N/A	No difference	Higher (g = 0.663, *p* = 0.005, *I*^2^ = 0.70)	Lower overall HRV in MDD (Hedges’ g = −0.301, *p* < 0.001); depression severity negatively correlated with overall HRV (r = −0.354, g = −0.131, *p* < 0.001, *I*^2^ = 0.46)
**Koch et al. (2019; [19])**	Adults without medication and current depression	21	2250/1982	none (excluded)	Lower (g = −0.318, *p* < 0.001, k = 13, *I*^2^ = 0.04)	Reduction (g = −0.462, *p* < 0.001, k = 9, *I*^2^ = 0)	Reduction (g = −0.195, *p* = 0.005, *I*^2^ = 0.26)	Higher LF/HF ratio (g = 0.195, *p* < 0.001, *I*^2^ = 0.44)	Reduction in HRV/IBI, VLF-HRV, SDNN
**Wu et al. (2023; [20])**	Adults with depression diagnosis and no somatic comorbidities, no antidepressant medication (24 studies in Chinese populations, 19 from other countries)	43	2359/3547	none (excluded)	Lower (g = −0.51, *p* < 0.001, *I*^2^ = 0.83)	Lower (g = −0.51, *p* < 0.001, *I*^2^ = 0.82)	Lower (g = −0.34, *p* = 0.002, *I*^2^ = 0.86)	No difference	Lower SDNN and PNN50 Subgroup analysis showed sig. lower PNN50 and LF-HRV in studies from China, but not studies from other countries
**Brown et al. (2018; [25])—clinical ^2^**	Late-life depression (mean age > 60 years), clinical studies	5	550/348	subgroup analysis of studies that either excluded current antidepressant users or reported unmedicated patients separately	No significant reduction	N/A	Lower (g = −0.626, *p* = 0.007, *I*^2^ = 0.61; g = −0.26, *p* = 0.001, *I*^2^ = 0.07, excluding one study)	N/A	Lower overall HRV (g = −0.334, *p* = 0.007) HF-HRV only numerically reduced (g = −0.331, *p* = 0.067)
**Brown et al. (2018; [25])—community**	Community-based samples	6	1526/12325	4/6 studies excluded antidepressant use/had an unmedicated subsample	No difference; significantly lower in unmedicated subsample (N = 1006) (g = −0.79, *p* = 0.039)	N/A	Lower (g = −0.128, *p* = 0.002, *I*^2^ = 0.42), significantly lower in unmedicated subsample (N = 1006) (g = −0.109, *p* = 0.003)	N/A	Lower overall HRV (g = −0.084, *p* = 0.042); no relationship with depressive symptom severity
**Koenig et al. (2016; [21])—case–control**	Children and adolescents	4	99/160	antidepressant use not excluded	Lower resting HF-HRV (g = −0.59, *p* = 0.01, *I*^2^ = 0.58)	N/A	N/A	N/A	N/A
**Koenig et al. (2016; [21])—continuous**	Children and adolescents	6	2625	NR	No significant association (HF-HRV: r = −0.041, *p* = 0.438, *I*^2^ = 0.75) with depressive symptom severity	N/A	N/A	N/A	N/A
**Baumeister–Lingens et al. (2023; [22])—case–control** (*update of Koenig* et al. *2016* [21])	Children and adolescents	9	608	antidepressant use not excluded	N/A	N/A	N/A	N/A	Lower vagally mediated (i.e., RSA/HF-HRV or RMSSD) HRV (SMD = −0.593, *p* = 0.046, *I*^2^ = 0.91)
**Baumeister–Lingens et al. (2023; [22])—continuous** (*update of Koenig* et al. *2016* [21])	Children and adolescents—mostly healthy and/or various psychiatric diagnoses	21	4224	NR	N/A	N/A	N/A	N/A	Significant association between vagally mediated HRV (RSA/HF-HRV/RMSSD) and depression severity (r = −0.077, *p* = 0.046, *I*^2^ = 0.91) moderated by sex: stronger negative correlations in samples with more females
**Chen et al. (2023; [23])**	Children and adolescents	10	410/409	mixed, antidepressant intake no exclusion criteria	Reduced (g = −0.38, *p* = 0.01, *I*^2^ = 0.59)	Reduced (g = −0.49, *p* = 0.01, *I*^2^ = 0.75)	No difference	N/A	Reduced PNN50 (g = −0.79, *p* < 0.01, *I*^2^ = 0.00), no difference for SDNN
**Ding et al. (2024; [24])**	Children and adolescents	31	4534	not explicitly excluded (not reported)	Negative correlation with symptom severity (r = −0.10, *p* < 0.001, *I*^2^ = 0.69); moderating effect of age, (coefficient = −0.03, *p* = 0.03)	Negative correlation with symptom severity (r = −0.18, *p* = 0.01, *I*^2^ = 0.78)	No correlation	N/A	No correlation of depression severity and SDNN Age-moderated HF-HRV relationship; stronger correlation above 12 years of age. No moderating effect of sex or other moderators

Abbreviations: HRV = heart rate variability; IBI = interbeat interval; HF-HRV = high-frequency heart rate variability; LF-HRV = low-frequency heart rate variability; VLF-HRV = very low-frequency heart rate variability; LF/HF ratio = ratio of low-frequency to high-frequency HRV; RMSSD = root mean square of successive differences; SDNN = standard deviation of normal-to-normal intervals; PNN50 = percentage of successive interbeat intervals differing by more than 50 ms; SMD = standardized mean difference; MDD = major depressive disorder; g = Hedges’ effect size; r = correlation coefficient; N = sample size, *I*^2^ = heterogeneity; % of variance ^1^. *1 effect sizes and *p*-values reported from table, but different values reported in original text. N/A = not reported. ^1^ Overall, heterogeneity within reported meta-analyses is high *I*^2^ > 50%. This complicates interpretation within meta-analyses and of findings between meta-analysis. Heterogeneity may be due to patient-related factors including age, sex, BMI, ancestry, etc. It is however notable that two meta-analyses, namely Koch et al. and Kemp et al., who both included patients with current depression and without antidepressant medication, show quite low rates of heterogeneity, particularly for HF-HRV, highlighting the important impact of antidepressant medication (see also section on antidepressant medication as a confounder and, in particular, the NESDA study). However, Wu et al. also excluded use of antidepressants and show high levels of heterogeneity. ^2^ Brown et al. [25] contrast with studies reporting reduced HF-HRV. This discrepancy may reflect differences in sample characteristics and/or respiration: because HF-HRV depends on breathing frequency, values can be biassed if respiration falls outside the conventional HF band. Monitoring respiratory rate is therefore especially important in older samples. Other explanations are possible.

**Table 3:** Summary of included longitudinal/observational, antidepressant/treatment-related, psychotherapy, and biofeedback studies examining associations between depression outcomes and heart rate variability (HRV).

Evidence Group	Study	Sample/Setting	Design	Treatment/Comparison	Depression Measures	Main Findings (HRV in the Context of Symptoms and Response)	Covariates/Adjustments/Controlled Variables (Pasted Text; Otherwise NR)	Limitations
**Longitudinal/observational**	Whitehall II study (2011 and 2016; [26,27])	Longitudinal cohort; >2200 participants over ~10.5 years	Longitudinal cohort, British civil servants aged 35–55	Pre–post comparison	30-item GHQ; depressive episode = ≥4 on GHQ depression subscale	Higher HRV → lower odds of incident depressive symptoms at FU only in men without baseline symptoms Baseline depressive symptoms not associated with HR/HRV at FU	Adjusted for age, ethnicity, civil service grade; physical activity, alcohol, smoking; CHD/heart failure, stroke, diabetes, hypertension, obesity (BMI ≥ 30); medication use (past 14 days)	no clinician-rated depression scale; limited episode history; civil servant sample; ECG equipment differed between waves
	Huang et al. (2018: twin study; [28])	146 male twins (73 pairs), 7-year FU	Baseline HRV → BDI over 7 years (cross-lagged within-pair)	Pre–post comparison	BDI-II + DSM-IV interview	Lower baseline HRV → worsening depressive symptoms longitudinally Bidirectional pathway observed, but AD use explained association of baseline depression → lower HRV at FU	Within-pair differences inherently control shared genes/family + testing environment; models adjusted for smoking, β-blocker use, education, alcohol, physical activity, CAD history; BMI, hypertension, diabetes; antidepressant use; also examined PTSD robustness; zygosity interactions	Male-only; relatively small cohort for number of covariates, no clinician-rated depression scale
	Yaptangco et al. (2015; [29])	336 young adults (psychology students)	Baseline HRV → depressive symptoms at 1-year FU	Pre–post comparison	BDI-II	Lower baseline HRV predicted depressive symptoms at 1 year	RSA + symptom stability, trait anxiety, BMI, meds, age	Restricted student sample; self-report depression, no clinician-rated depression scale, antidepressant use corrected only in some models
	An et al. (2020; [30])	464 community adults ≥ 65; antidepressant-free at baseline, 253 at FU	5-year longitudinal (cross-sectional + prospective)	Pre–post comparison	GDS	Baseline HRV ↔ later depression examined (direction/effect *p* NR)	Adjusted for baseline GDS, age, sex, education years, MMSE, CIRS (depending on model)	High attrition (~45%) patients who did not come back for FU had higher baseline GDS scores; selection bias; self-report depression scale, no clinician-rated depression scale; start of AD medication during follow-up not controlled for
**Antidepressant treatment/treatment-related longitudinal**	Licht et al. (2008: NESDA; [17])	2114 total; AD users *N* = 603, non-users *N* = 1511; 2-year FU	Longitudinal cohort	Pre–post comparison	IDS + CIDI	Starting Ads associated with decreased RSA RSA “recovered” after discontinuation	Age, sex, education; depression/anxiety severity (unclear if all models)	AD exposure definition may miss/overestimate medication effects or irregular use (requirement of 50% use over 1 month to be considered taking AD medication); small subgroups (e.g., new TCA only *N* = 12, TCA stopped *N* = 10)
	Kemp et al. (2010; [18])	18 articles; baseline 673 depressed + 407 controls; 186 with pre-post AD, free of antidepressant use at baseline	Meta-analysis	AD treatment (pre–post summarized)	DSM-III-R/DSM-IV/DSM-IV-TR diagnosis	Treatment with antidepressant medication (SSRI, Mirtazapine, Nefazodone) did not change overall HRV TCAs decreased HRV	Drug-naïve/washed out; age-matched controls	No meta-regression; limited sample; small sample to investigate various classes of Ads
	Hartmann et al. (2019; [31])	62 AD-free MDD vs. 65 controls baseline; 2-week FU after AD start	Case–control + 2-week longitudinal	AD initiation over 2 weeks	BDI-II, IDS-C, HDRS; MDD diagnosis	Baseline HRV differed by group Change in HRV correlated with change in symptom severity	AD-free at baseline; age, sex, heart rate	Small sample; short FU
	Brunoni et al. (2013: tDCS vs. sertraline; [32])	116 MDD; 6-week double-blind RCT; washout of prior antidepressant medication	Double-blind RCT	tDCS vs. sertraline	MINI; MADRS	No association: ΔMADRS with ΔRMSSD/ΔHF No responder–status association	AD-washout at baseline, matched by age/sex/cardiovascular risk/med status; covariate-adjusted models NR	No covariate model reported; small treatment arms
	Hage et al. (2017; [33])	66 MDD + 36 controls; 41 MDD at FU; no psychoptropic substances in last 4 weeks	12-week FU; two separate studies	Escitalopram vs. quetiapine XR, control comparison group	HAMD; BDI-II; DSM-IV MDD	Higher baseline RSA and LF-HRV in responders vs. non-responders No significant HRV change over time	Age, sex, BMI, ethnicity. Excluded other Axis I and II diagnoses, active suicidality, hypertension, dyslipidemia, diabetes mellitus, history of smoking or substance abuse (<6 months), and history of heart disease; no inflammations; female subjects not pregnant, lactating, or taking oral contraceptives	Small studies; two treatment conditions; no continuous symptom-change modelling, restricted smoking and comorbidities
	Chambers and Allen (2002; [34])	38 women treated: final N = 16 complete pre/post	Double-blind RCT; pre–post within treated sample	Acupuncture for depression	SCID DSM-IV; HRSD (31-item; reported standard 24-item); dHRSD subset	RSA increase correlated with HRSD decrease Baseline RSA not associated with baseline severity Responders had greater ΔRSA	Women only; age 18–45; extensive exclusions (other Axis I/II, endocrine/medical disorders, suicidal potential, pregnancy; left-handed excluded;	Restrictive sample: only ~42% retained
**Psychotherapy (CBT) intervention**	Neyer et al. (2021; [35])	50 psychosomatic inpatients (34 F/16 M); 68% on ADs; comorbidities common	Naturalistic inpatient pre-post; 6–12 weeks (mean ~9 weeks	Intensive inpatient psychotherapy (individual 5×/week + groups) + psychiatric care	HRSD-24 + BDI-II	Symptoms ↓ HRV indices (RMSSD, lnHF, etc.): no significant change Baseline HRV inversely related to baseline symptoms	Measurement controls (AM, no smoking/caffeine ≥ 3 h, etc.); subgroup checks smokers/BMI/sex; medication not controlled statistically (NR)	Naturalistic; medication not controlled
	Carney et al. (2000: CBT + stable CHD; [36])	30 depressed stable CHD + 22 nondepressed controls; depressed split mild vs. mod-severe	Pre-post depressed + nondepressed comparison; 4-month FU	Up to 16 CBT sessions; controls untreated	BDI; DSM-IV interview; remission classification	Severe group: 24 h/day/night HR ↓ Severe group: daytime rMSSD ↑ Mild depression + controls: largely unchanged	Excluded β-blockers + tricyclics + other autonomic meds; stable meds required; arrhythmia/high ectopy excluded; exercise recorded unchanged; covariate models NR	Stable CHD; small sample, no clinician-rated depression scale
	Carney et al. (2016: CHD + MDD (night HR/HRV predictors; [37])	157 enrolled; 124 with continuous ECG; remitters 64 vs. non-remitters 60	Baseline predictors of remission at 16 wks; FDA + ANCOVA	CBT up to 12 sessions/4 mo; sertraline added if insufficient response	DISH/HAM-D-17; BDI-II	significantly higher nighttime heart rates and lower nighttime VLF-HRV compared to remitters	Adjusted for age, sex, β-blocker, baseline AD, prior MI, BMI, smoking; tested HR/HRV × treatment interactions (ns)	No follow-up HRV; only report VLF (no LF/HF-HRV), artificially dichotomized (remission)
	Euteneuer et al. (2023; [38])	80 MDD (AD-free) + 40 matched controls	RCT; 14 weeks CBT vs. waitlist; 24 h ECG	14 week CBT vs. WL	SCID DSM-IV; BDI-II; MADRS	Overall HRV ↑ HF-HRV ns LF-HRV ns Moderation: higher baseline severity → larger CBT gains in daytime HRV indices	24 h blood pressure time-varying covariate; other covariates NR	Covariate set unclear
	Ayudhaya et al. (2022; [39]	82 AD-free older adults (≥60) with subthreshold depression; 2 HPHs; 41/group	9-mo single-blind cluster RCT	12 wk Behavioural Activation+usual care vs. usual care only	TGDS	SDNN improved vs. control: +7.59 ms (95% CI 1.67–13.50) lnHF improved: +0.44 (0.04–0.85) LF improved: +0.53 (0.09–0.98) LF/HF no difference	GEE adjusted for employment + education; standardized pre-test controls (no smoking/alcohol/caffeine ≥8 h; tested 8:30 am; talking, coughing, deep breathing, and body movements were controlled)	Small; subthreshold not clinical MDD, no clinician-rated depression scale, ultra-short-term (2.5 min segments) HRV analyses
	Chien et al. (2015: CBIBCRE; [40])	89 inpatients with MDD (43 exp, 46 ctrl) acute ward Taiwan	4 week Cluster-randomized repeated measures (baseline, week2, week4, FU)	4 week CBT-based group + breathing relaxation vs. control (3×/week, 60 min/session)	DSM-IV by physician; severity via BPRS;	HF-HRV ↑ in intervention group LF-HRV ↓: group difference; intervention significant LF/HF ↓ at follow-up vs. baseline	HRV model adjusted for age, SES, BPRS severity, psychiatric history; sex not modelled (mostly male)	Mostly male; attrition due to discharge; no dedicated depression scale
**Biofeedback interventions**	Donnelly et al. (2023: Meta-analysis HRV biofeedback; [41])	9 studies; N = 428 (HRVB 224; control 204)	Meta-analysis RCTs	HRVB vs. TAU/standard care	BDI-II/HADS/CES-D	Depressive symptoms improved: Hedges g = 0.478 HRV improved: g ≈ 0.223	ROB + publication bias tests; no covariate adjustment/meta-regression reported	Mixed conditions and protocols; possible publication bias for HRV outcome
	Caldwell et al. (2018: HRVB adjunct to psychotherapy; [42])	20 female MDD students (10 HRVB + TAU; 10 TAU) + 10 controls; 18–25	Randomized controlled adjunct design; baseline vs. 6 week FU	HRVB + psychotherapy vs. psychotherapy only	MINI; BDI-II	BDI-II: larger reduction in HRVB + TAU vs. TAU; SDNN: larger increase in HRVB + TAU vs. TAU, no change for HF-HRV and LF-HRV; Mediation: SDNN change partially mediated depression change (Sobel *p* = 0.03, one-tailed)	Measurement controls: no exercise/caffeine/tobacco ≥ 3 h; exclusion criteria as listed; covariate-adjusted models NR	Very small; no clinician-rated depression scale, antidepressant effect not included as covariate
	Lin et al. (2019: MDD + insomnia HRV-BF; [43])	48 MDD (24 HRV-BF, 24 controls), age 20–75, 3 hospitals	Matched case–control; 6 week pre-post; HRV-BF has 1-mo FU	HRV-BF weekly 60 min × 6 + standard care vs. standard care	DSM-5 MDD; BDI-II;	HRV-BF reduced depression compared to ctrl group SDNN ↑ and LF-HRV ↑ vs. controls; HF-HRV no significant change	Matched on sex + ±5 y age; ECG 9 am-5 pm; meds as usual; no caffeine/alcohol/smoking/excess exercise ≥ 3 h; meds not controlled statistically; post-test meds unknown	Variable testing timeslot; small sample; medication changes not tracked

Abbreviations: HRV = heart rate variability; HR = heart rate; FU = follow-up; GHQ = General Health Questionnaire; BDI-II = Beck Depression Inventory–II; GDS = Geriatric Depression Scale; IDS = Inventory of Depressive Symptomatology; CIDI = Composite International Diagnostic Interview; DSM = Diagnostic and Statistical Manual of Mental Disorders; HDRS/HRSD = Hamilton Rating Scale for Depression; HAM-D = Hamilton Depression Rating Scale; MADRS = Montgomery–Åsberg Depression Rating Scale; MINI = Mini International Neuropsychiatric Interview; SCID = Structured Clinical Interview for DSM; BPRS = Brief Psychiatric Rating Scale; TGDS = Thai Geriatric Depression Scale; CBT = cognitive behavioural therapy; CHD = coronary heart disease; MI = myocardial infarction; tDCS = transcranial direct current stimulation; RCT = randomized controlled trial; SES = socioeconomic status; MMSE = Mini-Mental State Examination; CIRS = Cumulative Illness Rating Scale; ECG = electrocardiogram; RSA = respiratory sinus arrhythmia; RMSSD = root mean square of successive differences; SDNN = standard deviation of normal-to-normal intervals; HF-HRV = high-frequency heart rate variability; LF-HRV = low-frequency heart rate variability; VLF = very-low-frequency HRV; TCA = tricyclic antidepressant; SSRI = selective serotonin reuptake inhibitor; GEE = generalized estimating equations; HPH = Health Promoting Hospital; TAU = treatment as usual; HRVB/HRV-BF = heart rate variability biofeedback. Up arrow = increased; Down arrow = decresed.

## Data Availability

No new data were created or analyzed in this study. Data sharing is not applicable to this article.

## Abbreviations

The following abbreviations are used in this manuscript:

**Abbreviation**	**Meaning**
HRV	Heart Rate Variability
IBI	Interbeat Interval
HF-HRV	High-Frequency Heart Rate Variability
LF-HRV	Low-Frequency Heart Rate Variability
VLF-HRV	Very Low-Frequency Heart Rate Variability
LF/HF ratio	Ratio of Low-Frequency to High-Frequency HRV
RMSSD	Root Mean Square of Successive Differences
SDNN	Standard Deviation of Normal-to-Normal Intervals
PNN50	Percentage of successive interbeat intervals differing by more than 50 ms
SMD	Standardized Mean Difference
MDD	Major Depressive Disorder
g	Hedges’ effect size
r	Correlation coefficient
CI	Confidence Interval
N	Sample size
k	Number of included studies
